# Reports of American Institutions for the Insane
*1. Of the Butler Hospital, for 1854.2. Of the Bloomingdale Asylum, for 1854.3. Of the New York State Lunatic Asylum, for 1854.4. Of the New Jersey State Lunatic Hospital, for 1854.5. Of the Pennsylvania Hospital for the Insane, for 1854.6. Of the Pennsylvania State Lunatic Hospital, for 1854.7. Of the Maine Insane Hospital, for the years 1854 and 1855.8. Of the New Hampshire Asylum for the Insane, for the years 1854 and 1855.9. Of the Vermont Asylum for the Insane, for the years 1854 and 1855.10. Of the Massachusetts Lunatic Hospital, Worcester, for the years 1854 and 1855.11. Of the Boston Lunatic Asylum, for the year 1852.12. Of the New York City Lunatic Asylum, for the years 1854 and 1855.13. Of the Maryland Hospital for the Insane, for the years 1853, 1854, and 1855.14. Of the Mount Hope Institution, for the years 1854 and 1855.15. Of the Western Lunatic Asylum, Virginia, for the years 1854 and 1855.16. Of the South Carolina Lunatic Asylum, or the years 1853 and 1855.


**Published:** 1857-04-01

**Authors:** S. Pliny Earle, Isaac Hays


### Art. IV.—REPORTS OF AMERICAN INSTITUTIONS FOR THE INSANE.*

BY S. PLINY EARLE.

(From the *American Journal of Medical Science.* Edited by Isaac Hays, M.D.)

The American reader who wishes to obtain a knowledge of insanity, its nature, its causes, and its proper treatment, without personal observation in an hospital, can hardly do better than procure a series of the Reports by Dr. Ray. We always shrink from them when preparing our notices, because of the labour required in the effort to do justice to both the author and our readers, and at the same time confine ourselves within justifiable limits.

| | Men. | Women. | Total. |
|---|---|---|---|
| Patients in the Butler Hospital, Dec. 31, 1853. . | 63 | 73 | 136 |
| Admitted in course of the year . . . . . . . . | 31 | 49 | 80 |
| Whole number . . . . . . . . . . . . . . . | 94 | 122 | 216 |
| Discharged, including deaths . . . . . . . . | 40 | 45 | 85 |
| Remaining December 31, 1854 . . . . . . . . | 54 | 77 | 131 |
| Of those discharged, there were cured . . . . . | ... | ... | 40 |
| Died . . . . . . . . . . . . . . . . . . . | ... | ... | 19 |

* 1. Of the Butler Hospital, for 1854.
2. Of the Bloomingdale Asylum, for 1854.
3. Of the New York State Lunatic Asylum, for 1854.
4. Of the New Jersey State Lunatic Hospital, for 1854.
5. Of the Pennsylvania Hospital for the Insane, for 1854.
6. Of the Pennsylvania State Lunatic Hospital, for 1854.
7. Of the Maine Insane Hospital, for the years 1854 and 1855.
8. Of the New Hampshire Asylum for the Insane, for the years 1854 and 1855.
9. Of the Vermont Asylum for the Insane, for the years 1854 and 1855.
10. Of the Massachusetts Lunatic Hospital, Worcester, for the years 1854 and 1855.
11. Of the Boston Lunatic Asylum, for the year 1852.
12. Of the New York City Lunatic Asylum, for the years 1854 and 1855.
13. Of the Maryland Hospital for the Insane, for the years 1853, 1854, and 1855.
14. Of the Mount Hope Institution, for the years 1854 and 1855.
15. Of the Western Lunatic Asylum, Virginia, for the years 1854 and 1855.
16. Of the South Carolina Lunatic Asylum, or the years 1853 and 1855.

The department for females has been so much crowded, that applicants for admission have been rejected.

| | |
|---|---|
| Patients admitted from 1848 to 1854, both inclusive . | 663 |
| Discharged, recovered . . . . . . . . . . . . . | 211 |
| Died . . . . . . . . . . . . . . . . . . . . | 121 |

The deaths are "equivalent to about 15 per cent. of the *average resident number of patients*,* one year with another."

"In 1850, when dysentery was very prevalent in this region, we had our share of it; but this year we had only a few cases, none of them being severe, and not more than three continuing beyond the third day. With these two exceptions, and that of an occasional influenza, we have been entirely exempt from epidemic disease."

"It is a noteworthy fact, that in most if not all our hospitals the mortality has been steadily increasing of late years." We have made no investigations in regard to that which is here asserted.

"*Premature removals*" is still one burden of complaint in the Reports from many of the institutions. "It is one of the disheartening experiences of our calling," says Dr. Ray, "to be so often obliged to see a patient removed just at the moment when it seemed as if our efforts to promote his recovery were about to be rewarded with success, though a longer perseverance would have been followed by no very obvious deprivations—none, certainly, which a year or two of restored health would not have fully repaired. Few things are more calculated to lower our estimate of human nature than *this balancing of reason, God's greatest gift to man, with a paltry sum of money*. . . . Few of our patients who fail to recover within five or six months are allowed to remain longer. It is concluded that everything has been done that we can do, and that the time has come for another experiment. And yet it appears, from one of Esquirol's tables, that in the French hospitals less than one-half of the recoveries occur within the first year. . . . I have no hesitation in saying that many of the incurables that form so large a share of the inmates of our hospitals, have been made so by the interference of well-meaning but injudicious friends."

From a very accurate exposition of the *secret*—if secret it may have been—in which lies the superior advantage of *hospital* over *domestic* treatment, we make an extract which will impart a sufficient idea of the whole:—

"Insanity implies the existence of bodily derangement, and therefore is a suitable object of medical treatment, which, of

* We regard this as the only accurate basis upon which to compute the mortality in any public institution.

course, would be more skilfully applied by men who are devoting their whole time and attention to this affection, than by those who observe it only on a very limited scale. But it also implies derangement of the ideas, hallucinations of the senses, perversion of the moral sentiments—all which, though the result of physical disorder, are, so far as their outward manifestations are concerned, in some degree under the control of others, and by such control—in a way not very well understood—the morbid process may be arrested. Now, it is the moral management prevalent in the hospitals of our time which so strongly distinguishes them from those of any former period, and determines, in a great measure, the amount of good which they accomplish. . . . It is one of their merits, indeed, that this management works so easily, and substitutes so quietly its own arrangements for the suggestions of disease, that the uninitiated observer finds it difficult to appreciate its real value, and thus often mistakes the character of its results. He sees the patient taking no medicine, perhaps; calm in his discourse and movements; readily complying with the wishes of others, and engaging, it may be, in some form of work or amusement; and he adopts the conclusion, which no opinion of the physician can shake, that the patient has recovered, or, at any rate, is so much better that he would do equally well at home, or in a private family. He can scarcely be made to believe that what he witnesses is chiefly the result of that special management peculiar to a modern hospital for the insane—of architectural arrangements which restrain without annoyance; of systematic regularity in the daily routine of life; of gentle manners, judicious firmness, vigilant, enlightened, and conscientious supervision. Now, these qualities are not a matter of accident, nor are they the growth of a day. They are the elaborated result of a profound study of the mental constitution, both in health and disease; of extensive inquiry into the various arts concerned in the erection and practical working of an extensive establishment, and of an organization of the service best calculated to effect its designed object."

"The peculiar restlessness of the insane, which impels them to roam about regardless of time and occasion, at the risk of their own safety and the peace of society, and which finds no sufficient restriction in the arrangements of an ordinary dwelling, short of confinement in a small apartment, is effectually controlled in an hospital; while the range of ample galleries and airing-courts prevents that control from being oppressive and unhealthy. Their fitful humours, their wild caprices, their impulsive movements, their angry looks, are met by the steady and straightforward will of attendants who have learned to perform their duty unbiassed by fear or favour. Having no object of their own to serve, imbibing the spirit of kindness which prevails around them, deterred from improper practices by a vigilant supervision, and aided by suitable architectural contrivances, they are enabled to manage their charge with the least possible degree of annoyance. Thus withdrawn from outward excitements, and especially from the persons and scenes connected with his mental disorder, the patient naturally becomes calmer, his mind opens to better suggestions, and finally seeks for repose in amusement or labour. And thus it happens that in many cases but little more is necessary to conduct the morbid process to a successful issue, besides giving the constitution a fair chance to exert its restorative powers unembarrassed by adverse influences."

In the Report of the Bloomingdale Asylum, Dr. Brown gives the following statistics :—

| | Men. | Women. | Total. |
|---|---|---|---|
| Patients in the Asylum, January 1, 1854 . . . | 56 | 68 | 124 |
| Admitted in course of the year . . . . . . . . | 58 | 64 | 122 |
| Whole number . . . . . . . . . . . . . | 114 | 132 | 246 |
| Discharged, including deaths . . . . . . . . | 64 | 55 | 119 |
| Remaining December 31, 1854 . . . . . . . . | 50 | 77 | 127 |
| Of those discharged, there were cured. . . . . | 22 | 26 | 48 |
| Died . . . . . . . . . . . . . . . . . . | 16 | 10 | 26 |

*Causes of Death.*—Old age, 6 ; exhaustive mania, 4 ; phthisis pulmonalis, 3 ; general paralysis, 3 ; epilepsy, 2 ; marasmus, 2 ; apoplexy, 1 ; disease of kidneys, 1 ; suicide, 1 ; use of opium, 1 ; "exhaustion from a suicidal attempt previous to admission," 1.

Of the six who died from old age, the youngest was 70, and one had reached the age of 95, preserving a remarkable degree of intellectual activity and genial humour within a few days of his departure.

"The insane, as a class," remarks Dr. Brown, "are unsound alike in mind and body. They inherit the multiform varieties of scrofula, and among them abound the Protean forms of nervous diseases, hysteria, chorea, neuralgia, and epilepsy. Some are victims to depraved appetites, unrestrained by an enlightened and vigorous will, and suffer the torments of alcoholic poison, which has paralyzed alike their physical energies and their moral sense. Cardiac, hepatic, renal, and uterine affections are common among them, excite and shape their delusions, and generally shorten their lives. Their sensations being enfeebled or perverted, they disregard extremes of heat and cold, and become indifferent to danger ; but while the mind may betray no indication of pain, their bodies suffer like those of sane men. Neglect of hygienic laws, and resistance to the regimen or habits imposed by others for their benefit, beget inevitable evils. Obstinate derangements of the digestive and assimilative organs are induced by prolonged abstinence, or excessive and unmasticated food; the circulation is languid from muscular inactivity; the extremities are cold and livid; slight abrasions of the skin become alarming ulcers; and serious visceral disease insidiously establishes itself, too often successfully resisting medical art. These patients become prematurely old; their intellectual perceptions and moral emotions disappear with healthy sensation; they sicken and die, often without an intimation of suffering, or an expression of concern.

"On the other hand, a remarkable exemption from prevailing epidemics is sometimes vouchsafed to these unfortunates. Thus, while cholera prevailed to a considerable extent in the neighbourhood of the Asylum during the past summer (1853), no instance of this disease occurred within our enclosure, and the whole household was preserved, in an unusual degree, from all affections of a similar character."

The long-desired improvement in the buildings of this institution has at length been accomplished by the enlargement of the "lodges," and the introduction into them of a system of forced ventilation, and heating by hot water. The beneficial effects of these have soon become apparent. "Quiet and sensitive patients have been effectually relieved of the annoying presence of the disorderly and noisy, and at the same time the latter have been permitted a degree of liberty formerly incompatible with the comfort of their associates."

In the means for ministering to all the physical comforts of its patients, the Bloomingdale Asylum has now but few equals in this country.

Dr. N. D. Benedict resigned the office of superintendent of the New York State Lunatic Asylum in June, 1854, and was succeeded by Dr. John P. Gray, who for some years had been connected with the institution as assistant physician. The report of the latter contains a large amount of interesting matter, from which we shall extract that which appears to be of the greatest value.

| | Men. | Women. | Total. |
|---|---|---|---|
| Patients in the Asylum, November 30, 1853 | 239 | 207 | 446 |
| Admitted in the course of the fiscal year | 191 | 199 | 390 |
| Whole number | 430 | 406 | 836 |
| Discharged, including deaths | 204 | 182 | 386 |
| Remaining November 30, 1854 | 226 | 224 | 450 |
| Of those discharged, there were cured | 98 | 66 | 164 |
| Died | 29 | 36 | 65 |

*Causes of Death.*—Phthisis pulmonalis 15, exhaustion from acute or periodic mania 11, exhaustion of chronic mania 5, old age and protracted mental disease 4, general paralysis 4, epilepsy 4, suicide 4, erysipelas 4, apoplexy 2, hemorrhoids 2, disease of liver 1, pneumonia 1, typhoid fever 1, chorea 1, not stated 7.

"In December last (1853), a case of smallpox occurred, which, though isolated as much as possible, was followed by others, and in the two months following we had twenty-three cases. By great precautions, the disease was confined entirely to the male department."

In the spring and summer of 1853, "the domestics engaged in the kitchens were seized with typhoid fever. We attributed this to the decayed state of the floors and timbers of the basement, and the decomposing impurities beneath." These causes being removed, there were no more cases of the disease.

One hundred and eight applicants for admission were rejected, and fifty-one patients discharged, for the purpose of receiving more recent and curable cases.

"During the past, as in former years, we have had many unhappy instances of injudicious haste in bringing patients to the asylum. One lady was admitted five days after her confinement; another before the burial of the child whose death was the immediate exciting cause of her disease, and many who were far too ill to travel."

"Thirty-four patients (eleven males and twenty-three females), have been admitted during the year with strong suicidal propensities. In several of these cases the ancestors had committed suicide, in two of them for three generations. In one male, it was impulsive; he was also homicidal, and left home at his own request, because he felt that the inclination to destroy his children, whom he loved tenderly, was gradually strengthening, while his power of resistance was growing weaker.

"All the epileptics (10) admitted, and the cases (6) of general paralysis, had either epileptic or intemperate ancestors."

Of the 390 patients received, the disease was directly hereditary in the numbers and manner shown in the subjoined table:—

| | Men. | Women. | Total. |
|---|---|---|---|
| From the paternal branch of the family . | 30 | 20 | 50 |
| From the maternal branch . . . . . . | 16 | 30 | 46 |
| From both paternal and maternal branches | 8 | 9 | 17 |
| Total . . . . . . . . . . . . . . . | 54 | 59 | 113 |
| Collateral predisposition manifested in the insanity of brothers, sisters, or cousins . | 10 | 19 | 29 |
| Numbers in which there was a family predisposition . . . . . . . . . . . . | 64 | 78 | 142 |

Thus, in 28·97 per cent. of the cases the disease was hereditary; and in 36·41 per cent. there were relatives insane. A larger number received the predisposition from the father than from the mother. This does not accord with the result of the researches of Dr. Baillarger, of Paris, which showed, as has been formerly mentioned in our notices, that, so far as the data which he had collected were concerned, a large majority received it from the mother.

"In one case included in the preceding table, the maternal great-grandmother, grandmother, mother, and two aunts, and the paternal grandfather, uncle, and two sisters, and one brother, have been insane. In another, the maternal grandmother, two uncles, and mother, the paternal grandfather and uncle, and two brothers, have suffered from attacks of insanity.

*Improper cases received.*—Typhoid fever, 1; chorea, 1; phthisis, 1; drunkards, 8; feigned insanity, to escape imprisonment, 2. Several, also, placed under the head of subacute mania, were simply cases in which there was mental prostration with tranquil delirium, the result of grave organic diseases. It is well known that when the brain and heart are seriously involved, there may be more or less prostration of the intellectual faculties; and when the lungs are implicated, often a state of exaltation not amounting to insanity. These persons were in such a feeble bodily state that we could not refuse them, although improper cases for admission, fearing they might die on their return home, as some lived in distant parts of the State. Most of them never left their beds after reception; some died in a few days, and others lingered for several weeks.

A patient received from one of the county poor-houses "had been for more than a year chained to the floor of his cell, and destitute of clothing and bedding, because *his habits were destructive and filthy.* He had lain upon straw, which was changed two or three times a week, and the person who attended him '*always carried a cow-hide, because he always attacked him, and he could only control him by whipping him.*' This man is now quiet and comfortable. Application has recently been made for the return of another, who, the superintendent of the poor states, 'has been in chains for months, and is in the most filthy and wretched condition.'"

Dr. Smith, of the Missouri Asylum, in his Report for 1854, discusses the question of the propriety of establishing separate asylums for the incurable insane, and, after giving his reasons for condemning the plan, says:—"I rejoice in the reflection that America has never been disgraced by one, and I trust that period will never occur." We, also, are opposed to such special institutions, but an asylum for incurables is not necessarily a disgrace to any nation. Some of those upon the continent of Europe stand, confessedly, in the foremost rank of establishments for the insane, and we have seen several of them which would not suffer in comparison with some of our asylums for both curables and incurables. Indeed, of the fifty-six *public* institutions for the insane which it has been our lot to visit, the very worst, with but a single exception, is in one of the original thirteen United States of America.

If our country has not been disgraced by an asylum erected particularly for the incurable insane, has she not been, is she not still disgraced by something even more objectionable? Read the several memorials of Miss Dix, embodying the results of her observations of the insane and their manner of treatment in the poor-houses, prisons, cellars, and specially-constructed cells in several of our states! Go to the numerous poor-houses, which have been, and still are, in effect, asylums for the incurable insane, with all the objectionable and none of the redeeming qualities of those to which Dr. Smith objects, and learn what is there to be learned. Or, simply, read the above extract from the report of Dr. Gray, and we may then ascertain whether our course, in the treatment of those who are alienate of reason, is such as to exempt us from reproach.

For ourselves, rather than allow the incurables to remain, as they are this day, we would rejoice to see them collected, to-morrow morning, into purely *technical* "asylums for the incurable." The establishments once in operation, the Argus eyes of trustees, of the Association of Medical Superintendents of Asylums for the Insane, of the communities in which they might be placed, and last, though far from least, of Miss Dix, so long as Heaven shall bless her with life and health, would watch them with a vigilance sufficient to prevent a large proportion of the abuses now heaped upon the unfortunate persons who would become the inmates of them.

| | |
|---|---|
| Patients admitted from January 16, 1843, to Dec. 1, 1854 | 4,313 |
| Discharged, recovered | 1,789 |
| Died | 511 |

"The *Opal* is still edited and published by the patients, and its proceeds devoted to their comfort and amusement. The profits of the last year, with the avails of a fair held by the ladies, amounted to four hundred dollars, which has been expended for books, improvements in the greenhouse, and in amusements. The *Opal* now receives about three hundred periodicals and newspapers in exchange."

The number of patients in the New Jersey State Lunatic Hospital, at Trenton, is as follows:—

| | Men. | Women. | Total. |
|---|---|---|---|
| Number of patients 1st Jan., 1854 | 98 | 107 | 205 |
| Admitted in course of the year | 56 | 67 | 123 |
| Whole number | 154 | 174 | 328 |
| Discharged, including deaths | 46 | 69 | 115 |
| Remaining at the close of the year | 108 | 105 | 213 |
| Of those discharged, there were cured | 25 | 32 | 57 |
| Died | 11 | 12 | 23 |

*Causes of Death.*—Consumption 4, general exhaustion 8, dysentery 8, epilepsy 1, apoplexy 1, asphyxia 1, congestion of the brain 1.

"During the months of August and September, a number of cases of dysentery occurred, several of which proved fatal.

"Besides the Museum and Reading Room," remarks Dr. Buttolph, "as a means of occupying and amusing the members of our household, we are now engaged in the erection of a calistheneum, or exercise room, twenty by sixty feet in extent, for the use of the female patients ; also, a ten-pin alley for the men. Both these structures are being erected by aid of contributions from various benevolent individuals, and will form very valuable means of promoting both physical health and mental tranquillity." The calistheneum is within the inclosed grounds for the women, and its estimated cost is one thousand dollars. Messrs. Morris, Tasker, & Morris, of Philadelphia, have contributed a "self-regulating hot-water furnace" with which to heat it, and several gentlemen, mostly in Newark, have given money to the amount of 675 dollars.

The laudable benevolence of many of our fellow-citizens is rapidly bringing some of the public establishments for the insane into a proper condition in regard to the facilities for curative treatment. As one extreme is often followed by its opposite, it may, perhaps, ere long, become a question whether there is not a danger that the desolation, the barrenness, and the wretchedness of the mad-house of the past, will be followed, in the Hospital for Mental Disorders of the future, by an objectionable and deleterious luxury.

Two additional wings to the Trenton Asylum are in progress. The house has been so much crowded by the number of patients during the year, that their proper classification has, at times, been impracticable. But a small part of the report of Dr. Buttolph is devoted to subjects which would particularly interest our readers.

"That which the skeleton is to him whose general form it represents, when in his prime of manhood," such must be our notice of the report for 1854, of the Pennsylvania Hospital for the insane.

We must fully coincide with Dr. Kirkbride in his remarks, that "because entire accuracy in every point may not be attained, it can hardly be urged as an excuse for not attempting to approach it; nor is it a sound reason for omitting all statistics, that wrong inferences and unfair comparisons have occasionally been made from some that were not entirely reliable. . . . I have never been able to discover a sound reason why tables of carefully recorded facts, or even of the opinions of intelligent physicians in reference to insanity, should not be just as important and reliable as if made in regard to other diseases."

| | Men. | Women. | Total. |
|---|---|---|---|
| Patients in the Hospital Dec. 31, 1853 | 112 | 123 | 235 |
| Admitted in course of the year . . | | | 178 |
| Whole number . . . . . . . . . . | | | 413 |
| Discharged, including deaths . . . | | | 190 |
| Remaining, December 31, 1854 . . . | 117 | 106 | 223 |
| Of those discharged, there were cured . | | | 98 |
| Died . . . . . . . . . . . . . | 15 | 11 | 26 |

*Causes of Death.*—Phthisis pulmonalis 6, acute mania 5, softening of the brain 4, epilepsy 1, paralysis 1, chronic bronchitis 1, acute inflammation of the bowels 1, dysentery 1, diarrhœa 1, pneumonia 1, acute dementia 1, disease of heart 1, hydrothorax 1, old age 1.

"There has been little acute disease of any kind—except of the brain—and no tendency to any of the summer or autumnal epidemics which prevailed in many parts of the country." The hospital has been full throughout the year, often crowded, and as many as fifty applicants for admission were not received.

| | Men. | Women. | Total. |
|---|---|---|---|
| Patients admitted since the opening of the hospital . . . . . . | 1384 | 1192 | 2576 |
| Discharged, cured . . . . . . . | 661 | 574 | 1235 |
| Died . . . . . . . . . . . . | 157 | 114 | 271 |

From two of the tables given in the report we derive the following in reference to the relative proportion of the different forms of mental disorder, and of their comparative curability:—

| | Admitted. | Cured. | Per cent. |
|---|---|---|---|
| Mania . . . . . . . . . . . | 1273 | 738 | 57.97 |
| Melancholia . . . . . . . . | 628 | 324 | 51.58 |
| Monomania . . . . . . . . . | 358 | 147 | 41.06 |
| Dementia . . . . . . . . . | 306 | 25 | 8.17 |
| Delirium . . . . . . . . . | 11 | 1 | 9.09 |

Of the 223 cases still remaining in the hospital, some will undoubtedly be restored to health, modifying, somewhat, these results.

The greater proportion of the annual reports of a public institution for the insane, pass, in each successive year, into the hands of a new set of readers. For this reason we have ever approved of the plan of entering into a more or less detailed account of the moral treatment in every report. To the constant reader this may become monotonous, but that effect is as nothing in comparison with the influence for good upon the minds of the people in regard to the true nature of these institutions, which cannot fail to be produced by such a course. In but few of the reports from our hospitals is so much space customarily devoted as in those of Dr. Kirkbride.

During nine months of the year, three entertainments in each week are given to the patients. "One evening is now devoted to scientific lectures, another to the exhibition of dissolving views, with interesting explanations of the scenery or pictures shown, and interspersed with music; and the third to music alone." On the other evenings of the week the teachers read to the patients in the wards. "The interest in these readings has been increased by the introduction of some pleasant exhibitions and music. The value of all this class of means can hardly be properly estimated by those who have not made a systematic trial of them, using them not, at long intervals, as a rare gratification, but frequently, as a regular part of the treatment. . . . . As a general rule, the more varied the pursuits of the patients, the more steadily they are kept employed in some way, the more comfortable they will be found to be, the better their general health and appearance, the more easily they may be managed, the less noise and excitement there will be in the wards, and the more efficient and valuable will be the services of those who are engaged in their care. No money expended in a hospital for the insane is better applied than when judiciously used in promoting these various objects."

A carriage-road on the pleasure-grounds for the men, similar to that on those for the women, is in progress. These roads will have a combined extent of a mile and three-quarters, and will be bordered with trees through most of the distance.

The Board of Managers of the Pennsylvania Hospital for the Insane, in view of the necessity for further accommodations, have determined to erect another establishment upon a tract of land containing seventy acres, immediately west of the pleasure-grounds of the present hospital buildings, "a site that cannot be surpassed for general adaptation and natural advantages, in a single tract, secured from encroachments by special legislative enactments." It is intended that it shall have "fixtures and arrangements of so superior a kind as, with the present buildings, to put our city (Philadelphia), in this respect, far in advance of any other on this continent, and to give to those who are mentally diseased advantages nowhere surpassed." The estimated cost is 250,000 dollars, and the buildings will be commenced when 150,000 dollars shall have been subscribed.

The report contains an elaborate memoir of Jacob G. Morris, late a Trustee of the Institution, who was on board the steamer "Arctic" at the time of her loss, and met, with so many others, an untimely death. As institutions for the insane have rarely, if ever, received such attention and sacrifice from a trustee or manager, we should fail of justice were we to omit the following extracts:—

"Possessed of an ample fortune, and with a truly benevolent heart, the activity, energy, and sound sense of Jacob G. Morris, made him a desirable manager in the charitable institutions abounding in our city. . . . . He made it a rule to expend all his income, whatever it might be; and as his own habits were in no way extravagant, a large portion of it was devoted to deeds of charity. . . . . He was strictly conscientious, and whatever he believed right he carried out steadily and fearlessly, without regard to what others might think of his course. . . . . He was elected manager of the Pennsylvania Hospital in September, 1844, and from that time took an active interest in all its departments. He was always ready to perform any services that were assigned him. . . . . He was a frequent visitor among our patients, and was always joyfully received. . . . . The slightest wish made by a patient, especially of the gentler sex, he rarely failed to esteem it a duty to gratify; and it was surprising what an amount of labour he would often undergo to enable him to effect it. It differed little with him whether it was to procure a toy for a child, patterns or materials for ladies' fancy work, a piano for a ward, a collection of books for the library, curiosities for the museum, or funds for some greater undertaking; he entered on the task with an equal zeal, and with an earnestness and hearty good-will, which never left a doubt of success. Rarely did he fail in anything he undertook; and his own gratification at such a result quite equalled that of those who were immediately benefited.

"He placed on our walls several valuable oil-paintings, and purchased expressly for the museum a large collection of curiosities; besides making numerous presents of a useful or ornamental character." . . . . When it was decided to erect the new hospital, as above-mentioned, "he at once offered 1000 dollars as his first contribution to the work, and devoted no small portion of the little time that elapsed before his departure for Europe, in calling attention to its importance and soliciting subscriptions in aid of the object. . . . . In every part of his travels he visited all the institutions for the insane worthy of note, and collected everything that he thought might be useful in our new undertaking. In the very last letter I received from him, this subject was earnestly dwelt upon, and, although anxious to be at home, he declared his willingness to return to Europe at any time as one of a commission to visit the best foreign institutions, defraying his own expenses, and contributing liberally to those of his associate." Truly, the Pennsylvania Hospital has met with a great, it is to be feared an irreparable, loss!

The report of Dr. Curwen, of the Pennsylvania State Lunatic Hospital, furnishes the following statistics:—

| | Men. | Women. | Total. |
|---|---|---|---|
| Patients in the Hospital December 31, 1853 . | 99 | 83 | 182 |
| Admitted in course of the year . . . . . | 93 | 51 | 144 |
| Whole number . . . . . . . . . . . . | 192 | 134 | 326 |
| Discharged, including deaths . . . . . . | 65 | 47 | 112 |
| Remaining, December 31, 1854 . . . . . | 127 | 87 | 214 |
| Of those discharged, there were cured . . . . | .. | .. | 29 |
| Died . . . . . . . . . . . . . . . . | .. | .. | 22 |

*Causes of Death.*—Exhaustion of chronic mania 10, general paralysis 4, peritonitis 2, bilious fever 2, inflammation of brain 2, consumption 1, dysentery 1.

"In common with the surrounding section of country, our household was visited, during the latter part of summer and through the autumn, with intermittent and remittent fevers, in many cases of a very obstinate form, and of a very irregular character. The diseases were clearly attributable to an epidemic constitution of the atmosphere, as most of those attacked were, as far as possible, removed from the usual exciting causes of those diseases."

In discussing the etiology of mental disorders, Dr. Curwen maintains that no exciting cause will produce insanity, unless the nervous system be in a favourable condition for its development, and that that which is often assigned as the *cause* in individual cases, as, for example, the reading of the Bible, is rather the *effect* of previous mental depression dependent upon the abnormal condition of the body.

"In those who are natives of other countries, a different class of causes, to a certain extent, acts. The disappointment of their hopes, so fondly cherished and often so rudely crushed, the entire change of climate and mode of life, the difficulty of obtaining employment and support for their families, with the too frequent resort to intoxicating drinks, all contribute their share to bring on that condition of the system which ends in insanity; and the natural elasticity of the system appears to be so far destroyed that, in a large majority of cases, it never fully regains its former tone and vigour."

By the report of Dr. Harlow, of the *Maine State Asylum*, it appears that at that institution, on the 30th of November, 1853, the number of

| | Men. | Women. | Total. |
|---|---|---|---|
| Patients was . . . . . . . . . | 61 | 58 | 119 |
| Admitted in course of the year . . . | 57 | 53 | 110 |
| Whole number . . . . . . . . . . | 118 | 111 | 229 |
| Discharged, including deaths . . . | 56 | 58 | 114 |
| Remaining, November 30, 1854 . . | 62 | 53 | 115 |
| Of those discharged there were cured . | 26 | 23 | 49 |
| Died . . . . . . . . . . . . . | 16 | 16 | 32 |

*Causes of Death.*—Dysentery 16; general paralysis 5; consumption 2; old age 2; marasmus 2; serous apoplexy 1; congestion of brain 1; typhoid fever 1; gangrene 1; epilepsy 1.

"About the 1st of August," says the report, "an *epidemic diarrhœal dysentery* broke out in our wards, and for three months little else than the sick and dying occupied our attention. There was scarcely an individual connected with the hospital family who escaped the ravages of the disease. Officers, attendants, nurses, and assistants shared alike with the patients in the attack. Just in the midst of the epidemic, when it would seem the services of the superintendent and steward were most needed, we were prostrated and unable to perform our duties. Having no medical assistant, we were obliged to call in a neighbouring physician to attend the patients. Fortunately, the trustees were able to procure the valuable services of ex-Governor Hubbard, who was formerly, for several years, a member of their board, and who has always felt and taken a deep interest in the hospital. He visited us daily for four weeks, and attended upon all the sick in the house till we were able to attend to our duties." Of ninety persons attacked by this epidemic, seventeen, of whom sixteen were patients, and one, a female attendant, died.

The number of patients admitted, since the first opening of the hospital for their reception, is 1430. Discharged, 1316, of whom 590 had recovered. Died 175.

To avoid the labour and the inconveniences attendant upon the return of patients who, on the supposition of recovery, have left the hospital with the formalities of a regular discharge, but who, after a few days, either suffer a relapse or give evidence of imperfect restoration, Dr. Harlow has adopted the plan pursued at some of the European hospitals, of discharging all in regard to whom he has "doubts of their fitness, on trial, for a period of two weeks."

Of 1200 *patients* who have been in the hospital, 586 had insane relatives. It is not stated whether these 1200 were so many *persons*, or merely so many *cases*, including a considerable number of re-admissions of the same person—conditions which materially affect the percentage.

In some remarks upon the deleterious effects of a forced early *intellectual* education, Dr. H. remarks that he "was most forcibly struck, in reading an account of a class of students who graduated at one of our New England Colleges, in 1827. It was found that of this class, numbering twenty-three, all but two had survived the lapse of a quarter of a century; and it was also found that nearly every member of the class had arrived at adult age before entering college, thus escaping that premature excitement and development of the intellect which paves the way to mental disease, and furnishes tenants for many an early grave."

At the date of this report an additional wing for females was in course of construction.

In the report for 1855 we are informed that the new wing is completed, and occupied. The original design is thus finished, and the hospital can now accommodate two hundred and fifty patients.

| | Men. | Women. | Total. |
|---|---|---|---|
| Patients in the Hospital, Nov. 30, 1854 . . . | 64 | 51 | 115* |
| Admitted in course of the year. . . . . . . | 66 | 62 | 128 |
| Whole number . . . . . . . . . . . . . . | 130 | 113 | 243 |
| Discharged, including deaths . . . . . . . | 44 | 44 | 88 |
| Remaining, November 30, 1855 . . . . . . | 86 | 69 | 155 |
| Of those discharged, there were cured . . . . | | | 41 |
| Died . . . . . . . . . . . . . . . . | | | 19 |

Deaths from general paralysis 5, epilepsy 3, chronic diarrhœa 3, tubercular consumption 2, congestion of the brain 3, old age, nephritis, and typhoid fever, 1 each.

"The propensity," says Dr. Harlow, "that exists in the minds of many of the friends of our patients, to remove them from the hospital too soon after they have been admitted, continues to be an evil which we should be glad to see eradicated. We are happy to say, however, that the evil appears to be growing less from year to year."

| | |
|---|---|
| Patients admitted since the Hospital was opened . . | 1559 |
| Discharged recovered . . . . . . . . . . . . | 631 |
| Died . . . . . . . . . . . . . . . . . . . | 193 |

"Owing to the two epidemics, and the great calamity by fire, with which the hospital has been visited since its existence, the bill of mortality is larger than (that of) some similar institutions."

* The number assigned to each sex does not correspond with that of the report of the preceding year.

Basing his calculation upon the results obtained by the commission which took the census of the insane and the idiots in Massachusetts, in 1854, Dr. Harlow concludes that, in Maine, there are 1365 lunatics and 560 idiots. "The question arises," he remarks, "where are all these 1365 lunatics, and what is their condition? Some are cared for at home by their friends, either chained or caged, if unmanageable, and some 150 are in the hospital, while by far the largest proportion of them are at the various Almshouses in the State, many of them caged and chained, because they can be kept a few cents less per week than it costs at the hospital." Where is that *other* "Maine Law?"

At the New Hampshire Asylum for the Insane, on the 31st of May, 1853, the number of

| | Men. | Women. | Total. |
|---|---|---|---|
| Patients was . . . . . . . . . . | 70 | 73 | 143 |
| Admitted in the course of the year . | 72 | 69 | 141 |
| Whole number . . . . . . . . . . | 142 | 142 | 284 |
| Discharged, including deaths . . . | 67 | 56* | 123 |
| Remaining, May 31st, 1854 . . . | 77 | 84 | 161 |
| Of those discharged, there were cured | 34 | 29 | 63 |
| Died . . . . . . . . . . . . | 7 | 7 | 14 |

"During the whole year our household has enjoyed remarkable physical health. We have been entirely exempt from epidemics of all sorts, and acute disease has been almost unknown. Cleanliness, regularity of life, and a most healthful location have been the chief causes of this desirable state of things. The deaths which have occurred, with a single exception, were of those who for a long time were considered incurably insane, and who at last were literally worn out by the continued and unremitting force of their malady.

"Through the whole year our female halls have been full, and often crowded, and our male halls at all times crowded." This condition "prevents a proper classification of patients, and seriously interferes with all curative measures." Dr. Tyler therefore recommends that an additional wing be erected.

"We can in almost no case infallibly pronounce a person incurably insane; certainly the records for the year show the recovery of some whose improvement seemed impossible, and whose present condition, among their friends, and in perfect health and soundness of mind, seems a miracle.

"The house is now lighted with gas, and we not only find its use more convenient, comfortable, and cleanly than oil, but its

* These numbers are quoted from the report. But if 67 men and 56 women were discharged, the number remaining would be 75 and 86, instead of 77 and 84.

brilliant light a curative means, in making our previously half-lighted halls cheerful and pleasant."

Report for 1855 :

| | Men. | Women. | Total. |
|---|---|---|---|
| Patients on the 31st of May, 1854 . | 77 | 84 | 161 |
| Admitted in course of the year . . . | 45 | 40 | 85 |
| Whole number . . . . . . . . | 122 | 124 | 246 |
| Discharged, including deaths . . . | 50 | 41 | 91 |
| Remaining, May 31, 1855 . . . . | 72 | 83 | 155 |

Among the patients who were discharged recovered, Dr. Tyler says there was " a man of intelligence and education who was for nearly eleven years an inmate of this institution."

The asylum is rapidly becoming filled with incurables. But about one-half of the applicants for admission can be received. The doctor recommends that an additional wing be erected. A building for the violent patients is in progress.

From the answers to circulars sent to every city and town in New Hampshire, and from other sources, Dr. Tyler ascertains that there are thirty-five insane persons belonging to the State who " are supported by their friends or guardians in hospitals in other States; and that there are now resident in the State more than 550 insane persons, only 155 of whom are in this asylum. Of the remainder, many are kindly and comfortably taken care of at home, or with friends, or at almshouses ; but others are chained, and caged, and sadly neglected ; in filth, and exposure to the inclemencies of the weather. Some instances of cruelty and neglect have lately come to our knowledge, that, if known, would startle the neighbourhoods in which they have occurred."

| | |
|---|---|
| Whole number of patients from the opening of the Asylum | 1284 |
| Discharged cured . . . . . . . . . . . . . . . | 547 |
| Died . . . . . . . . . . . . . . . . . . . . . | 118 |

At the Vermont Asylum for the Insane, on the 1st of August, 1853, the number of

| | Men. | Women. | Total. |
|---|---|---|---|
| Patients was . . . . . . . . . . | 183 | 189 | 372 |
| Admitted in course of the year . . . | 77 | 86 | 163 |
| Whole number . . . . . . . . . | 260 | 275 | 535 |
| Discharged, including deaths . . . | 72 | 74 | 146 |
| Of whom there were recovered . . . | | | 80 |
| Died . . . . . . . . . . . . | | | 40 |

It appears that a large number of the patients at this Asylum are employed on the farm, in the garden, and in the workshop. Dr. Rockwell also encourages them to join in "all amusements which require exercise of the body as well as diversion of the mind, and especially riding, walking, playing billiards, ten-pins, quoits, and the like." He would "rather they would play chess, draughts, cards, and such like games than do nothing."

| | Men. | Women. | Total. |
|---|---|---|---|
| Patients, August 1st, 1854 . . . . . | 188 | 201 | 389 |
| Admitted in course of the year . . . | 78 | 86 | 164 |
| Whole number . . . . . . . . . . | 266 | 287 | 553 |
| Discharged, including deaths . . . | | | 159 |
| Remaining, August 1st, 1855 . . . | | | 394 |
| Of those discharged, there were recovered | | | 79 |
| Died . . . . . . . . . . . . . | | | 52 |
| Whole number of patients since the opening of the Asylum . . . . . | | | 2393 |
| Discharged, recovered . . . . . . . | | | 1127 |

The unusually large number of deaths during the last year, is accounted for, in part, by the prevalence of a severe and fatal form of dysentery, which appeared among the patients in the early part of summer, and continued with unabated severity throughout that season. The number of attacks is not mentioned, neither is that of the cases in which it proved fatal. Warned by the two epidemics which have been mentioned, the directing authorities of the institution have ordered the construction of two infirmaries, one for either sex, wherewith, in the event of a future similar invasion of disease, the invalid patients can be isolated from the others. These apartments have been commenced.

The State Lunatic Hospital of Massachusetts, at Worcester, has for several years been greatly, almost unjustifiably crowded with inmates. It, its officers, and its patients have at length obtained some relief. The inconvenient and unwholesome condition of things has been changed for the better. Another State Hospital has been erected at Taunton. To this, on the 7th of April, 1854, "and on each of the five succeeding Fridays, a *car load* of patients" were transferred from the hospital at Worcester. The number thus removed was 210. No accident occurred in this rarely-paralleled migration. "The patients were mostly of a very orderly class, and they were gratified with the ride. Notwithstanding this great abstraction from its wards, the hospital was left "quite full, but not crowded," and it became possible to abandon the use of a number of cells and improperly-contrived rooms which had long been tenanted by patients.

| | Men. | Women. | Total. |
|---|---|---|---|
| Patients in the Hospital, Dec. 1, 1853 | 266 | 254 | 520 |
| Admitted in course of the year . . . | 125 | 174 | 299 |
| Whole number . . . . . . . . . | 391 | 428 | 819 |
| Discharged, including deaths . . . | 198 | 240 | 438 |
| Remaining, Nov. 30, 1854 . . . . | 193 | 188 | 381 |
| Of those discharged, there were cured | 45 | 77 | 122 |
| Died . . . . . . . . . . . . . . | 15 | 19 | 34 |

*Causes of Death.*—Marasmus 5 ; consumption 4 ; lung fever 4 ; maniacal exhaustion 7; apoplexy and palsy 3; epilepsy 2; erysipelas 2; suicide, dropsy, chronic dysentery, diarrhœa, congestive fever, asthma, and jaundice, 1 each.

In connexion with the causes of insanity, Dr. Chandler makes the following remarks:—"Probably in no part of the world are the causes of insanity more numerous and more active than among the population of Massachusetts. Here the mind, and body too, are often worked to the extreme point of endurance. Here wealth and station are the results of well-directed efforts; and the general diffusion of intelligence among the people stimulates a vast many of them to compete successfully for these prizes. But in the contest, where so many strive, not a few break down. The results on their minds may not, perhaps, be any less disastrous, whether wealth and station are obtained, or not. The true balance of the mind is disturbed by prosperity as well as by adversity. It is only in a sound body that the manifestations of the mind are sane and entirely healthy. As a people, we cannot boast of the highest standard of physical health, although we may of general intelligence, enterprise, and hard work."

In the course of the year 1855, very important improvements were made in this establishment. Not the least of these was the introduction of the apparatus for heating by steam, and that for ventilating by mechanical power. Relieved, by the hospital at Taunton, of its great excess of patients, and brought by improvements, more nearly into conformity with the idea of the times, this institution may still, for a long number of years, continue its career of usefulness. Indeed, it seems that Dr. Chandler is unwilling to acknowledge that it has ever merited the impression which has been made upon the public mind in regard to it. "Whatever," he remarks, "may have been said against this hospital—and most of what has been said about its defects, has been so said as an excuse to make it still better—it has always afforded, and does now, with all the progress made in others, a residence as comfortable and as cheering, and as healthful to the patient, as any similar institution in this country."

In this expression we believe Dr. Chandler is mainly correct. The only exception suggested relates to the hygienic condition of the building. It can hardly be assumed that, with its comparatively low ceilings, and its imperfect ventilation, it can be so healthful, other things being equal, as some of the similar edifices more recently erected. For the impression which has gone abroad in regard to it, the Board of Trustees who have the control of it are chiefly responsible. They drew the picture of its defects; and if their painted grapes bear such a resemblance to reality that the birds have pecked at them, truly it is not the birds that should bear the blame. All who have visited the hospital are well aware that the picture was a sketch of the shady side alone, and that another, drawn from the sunny side, might be made as attractive as the first was repulsive.

From its earliest years we have been a not unfrequent visitor to this hospital, and in this place we feel bound to acknowledge our belief, that from its origin, it has been not only *well*, but *very* well managed. We would shun invidious distinctions—we shall make none; yet justice demands from us the expression, that there is no institution in the country at which we could, at any time, have placed a friend with greater confidence that all his necessities would be supplied; that his comforts would be carefully ministered to; that he would be shielded from abuse; and that his restoration would be wrought for with watchful care, with constant assiduity, and with that skill which is the result of a good professional knowledge, combined with practical experience.

"We avail ourselves of this occasion," write the trustees, in their report, "to bear testimony to the fidelity and signal ability with which Dr. Chandler has discharged the duties of his position, and to the great success which has attended his labours during the whole period of his superintendence."

Dr. Chandler has resigned his office, and Dr. Merrick Bemis, for some years one of the assistant physicians of the hospital, has been appointed as his successor.

| | Men. | Women. | Total. |
|---|---|---|---|
| Patients in the Hospital Dec. 1, 1854 | 193 | 188 | 381 |
| Admitted in the course of the year . | 86 | 113 | 199 |
| Whole number admitted in the course of the year . . . . . . . . . | 279 | 301 | 580 |
| Discharged, including deaths . . . | 111 | 133 | 244 |
| Remaining, Nov. 30, 1855 . . . . | 168 | 168 | 336 |
| Of those discharged, there were cured | 50 | 59 | 109 |
| Died . . . . . . . . . . . . | 13 | 14 | 27 |
| Admitted from Jan. 18, 1833, to Nov. 30, 1855 . . . . . . . . . . | 2451 | 2505 | 4956 |
| Discharged, recovered . . . . . . | 1089 | 1195 | 2284 |
| Died . . . . . . . . . . . . | 281 | 272 | 553 |

Of this aggregate number of deaths, 87 are attributed to marasmus; to "consumption 67; apoplexy and palsy 59; maniacal exhaustion 59; epilepsy 50; suicide 22; lung fever 22; disease of the brain 21; disease of the heart 20; diarrhœa 19; erysipelas 17; old age 14; typhus fever 11; dysenteric fever 10; dropsy 10; inflammation of the bowels 8; hæmorrhage 6; cholera morbus 5; chronic dysentery 5; gastric fever 5; cholera 4; mortification of the limbs 3; from intemperance 3; bronchitis 3; congestive fever 3; hydrothorax 3; convulsions 2; asthma 2; disease of the bladder 2; cancer 2; jaundice 2; land scurvy 1; concussion of the brain 1; fright 1; rupture 1; pleurisy 1; chorea 1."

In reference to the salubrity of the hospital, this schedule of the mortality among almost five thousand patients, in the course of a period but little less than twenty-three years, is well worthy of a careful perusal. Its testimony is more reliable than that of individual opinion; more forcible than arguments deduced from theories of architectural construction. The almost entire exemption from fatal epidemics, from severe endemic fevers, and from other acute diseases, to which it bears record, will find but few parallels in any other institution of the kind in any quarter of the globe.

Under the table of "Causes," we find the following remarks:—

"Spiritualism of the present day is of the last (moral) class of causes. This singular mental phenomenon has for some years engaged a part of the minds in this vicinity, and some few cases have been brought to us, the past as well as previous years, arising, it was supposed, from investigating its phenomena, and from believing in its supposed truthful revelations of the future state of existence. If it was true that this process of investigation did really open to the mind any knowledge of the world to come, not revealed to us by the Scriptures—which many of its votaries assert and believe—then it would be a cause calculated in the highest degree to engage, excite, and disturb the mind. But it has been said by those best prepared to investigate closely, that the responses through the mediums contain no ideas of this or the next world, that were not then, or had not previously been, in the mind of some one present. It may be a new faculty of the mind, but its field of operation lies this side of the grave."

In regard to moral treatment, Dr. Chandler writes as follows:—"We recognise the principle of giving the largest liberty, and the greatest freedom from restraint, in each case, consistent with the security of the individual and safety of the community. . . . About one-half of our patients perform some kind of labour, more or less useful. For plain work, the patients are ready and very efficient. One day this autumn the patients, with one hired man, dug, took off the tops, and put into the cart, 480 bushels of carrots. Some are ingenious mechanics. Two have assisted the carpenters most of the season. One gentleman has made all the soft soap—350 barrels a year—and some of the bar soap, for five or six years. He has gathered the materials, made and distributed, and attended to the economical use of it. No one can make better soap than he. Two have made our baskets for years, and have supplied themselves, in part, with clothing. One female has made pantaloons and vests to supply the demand in our family. The females make the shirts, and knit the stockings; they wash and mend; they cook, and they help about all our domestic affairs. . . . . Whip-lash braiding . . . is the best employment I can think of to introduce among our patients in-doors."

The Report for the fiscal year ending with the month of November, 1852, is the latest which we have seen from the Boston Lunatic Asylum:—

| | Men. | Women. | Total. |
|---|---|---|---|
| Patients at the beginning of the year | 100 | 141 | 241 |
| Admitted in course of the year | 41 | 11 | 52 |
| Whole number | 141 | 152 | 293 |
| Discharged, including deaths | 34 | 15 | 49 |
| Remaining at end of year | 107 | 137 | 244 |
| Of those discharged, were cured | 17 | 5 | 22 |
| Died | 14 | 8 | 22 |

*Causes of Death.*—Consumption, 8; debility, 3; general paralysis, 3; epilepsy, 2; chronic mania, 2; smallpox, 1; "Asiatic diarrhœa," 1; old age, 1; marasmus, 1.

"It is a noticeable fact," writes Dr. Walker, "that no death from dysentery has occurred. This disease has prevailed extensively among us for several successive years, always bringing with it great suffering and ceaseless anxiety. Early in August it appeared in a very violent form, bringing several of our household rapidly to the verge of the grave. Fires were immediately lighted in the furnaces morning and evening, so that when the patients were rising and retiring a current of warm air should be passing through the halls and bedrooms. . . . . The most unpromising cases speedily began to amend, and at the time when the disease usually raged the most fiercely, not a case was under treatment."

The Report for 1851, from this institution, contained a brief but interesting account of the case of an Irish boy, among the patients. This account was, in whole or in part, transferred to our notices. The Report before us states that the boy has left the institution "giving promise of future usefulness."

New York City Lunatic Asylum, on Blackwell's Island:—

| | Men. | Women. | Total. |
|---|---|---|---|
| Number of patients on the 1st of January, 1854 | 232 | 310 | 542 |
| Admitted in course of the year | 224 | 262 | 486 |
| Whole number | 456 | 572 | 1028 |
| Discharged, including deaths. | 211 | 262 | 473 |
| Remaining, December 31, 1854 | 245 | 310 | 555 |
| Of those discharged, there were cured | .. | .. | 186 |
| Died | .. | .. | 190 |

Among the cases cured, there were four of delirium tremens. Of the cases admitted, nine were improper subjects for the institution.

*Causes of Death.*—Cholera, 83; consumption, 34; paralysie générale, 19; typhomania, 9; "debilitas," 9; epilepsy, 8; congestion of brain, 7; dysentery, 3; chronic diarrhœa, 3; old age, 2; typhus fever, pneumonia, erysipelas, hydrocephalus, albuminuria, suicide by suspension, injuries of head, injuries from fall, "submersion," peritonitis, gastroscirrhus, pericarditis, ascites, 1 each.

Dr. Ranney gives the following account of the cholera, by which, as will have been perceived, nearly one-half of the mortality was occasioned:—

"The epidemic commenced on the 22nd of July, and terminated on the 22nd of August. An attendant, however, was attacked on the 11th of September, and died in twelve hours. Four of the other attendants had cholera of a severe form; but all recovered. It seemed more violent and proved more fatal than in 1849, and nearly the same class was affected—viz., those n whom the constitution was greatly impaired from chronic disease, and the mind reduced to the most hopeless state. Frequently, the first warning was complete collapse, characterized by blueness of the skin, coldness of the surface, and loss of pulse. Cramps were less common than in 1849. If diarrhœa occurred, as a premonitory symptom, it was readily checked by medicine.

"Chronic diarrhœa has become much less frequently a cause (of death) since the introduction of Croton water on the island."

Of the 486 patients admitted in the course of the year, only 97 were natives of the United States. Of the foreigners, 241 were from Ireland, 91 from Germany, 21 from England, and 9 from Scotland; the remainder were from various countries. 100 were supported by the Commission of Emigration, and all these were emigrants of the preceding five years.

"A large proportion of the recent emigrants recover, the derangement of mind being generally produced by privations on ship-board, and the changes necessarily incident on arriving in a strange land. Their exposures and sufferings are occasionally very great in crossing the Atlantic, and, in a few, the aberration of intellect has seemed to depend entirely on the want of sufficient nourishment. A poor German boy was admitted last March, who had just arrived in New York. His suffering from starvation had been so great as to obliterate from his memory all knowledge of having crossed the ocean, and he fancied himself in his fatherland! He would implore me, in the strongest terms, to allow him to go on his journey, as in a few hours he would meet his father and mother, who were anxiously awaiting his return. Then a change would come over him, and he would imagine that he was detained as a culprit. He would plead his innocence with feeling eloquence, and in the most melting terms. These delusions were so firmly fixed that he would listen to no explanation, and the only effectual quietus was the liberal and constant supply of nutriment. His thirst fully equalled his appetite for food. I subsequently learned that he was a native of the Grand Dukedom of Baden, and that he had been seventy days in making the voyage from Bremen to New York. In two weeks the delusion disappeared, and he became fully conscious of his condition. In two months his mind was perfectly restored, when he left the asylum, as noted for excessive fatness as he had previously been for his emaciated and meagre appearance."

We rejoice to learn, as we do from the report for 1855, that one of the foulest blots which has rested upon the practical psychiatry of our country, has at length been effaced. "The most decided improvement ever made in this asylum," remarks Dr. Ranney, "has been consummated the past year. I refer to the entire removal of prisoners, not only from their immediate connexion with the insane, but from the institution.

"From 1826 to 1847, the work of the asylum was performed, and the principal charge of its inmates taken by persons transferred from the different penal institutions on the island. At the commencement of the year last named, six of that class were employed in each of the halls, and between fifty and sixty engaged as domestics about the building. One-fourth of the whole number at the asylum being convicts, the institution differed little, in its *morale*, from a prison. It was urged upon the common council, 'that the same individuals who were committed in the city as criminals, and required an armed keeper in the penitentiary, were sent here to take charge of a class who require the most mild and soothing treatment.' But the memorials sent soliciting a change in the system, produced no effect."

And be it remembered that no action was taken upon the subject until after the asylum had ceased to be one of the footballs of partisan politics, by that worthy act of the State Legislature, which wrested the government of the Almshouse Department of the City of New York from the municipal authorities, and vested it in a Board of Governors selected in equal, or nearly equal numbers, from the two most prominent political parties of the day. The change commenced in 1850, when prisoners were removed from three of the halls for patients, and has gradually progressed to its final completion.

| | Men. | Women. | Total. |
|---|---|---|---|
| Patients in the Asylum Dec. 31, 1854 | 245 | 310 | 555 |
| Admitted in course of the year . . | 163 | 208 | 371 |
| Whole number . . . . . . . . . . | 408 | 518 | 926 |
| Discharged, including deaths . . . . | 170 | 183 | 353 |
| Remaining, December 31, 1855 . . | 238 | 335 | 573 |
| Of those discharged, there were cured . | | | 200 |
| Died . . . . . . . . . . . . | | | 100 |

The disease of six patients, recorded among the cured, was *delirium tremens.*

Deaths from consumption 29 ; paralysie générale 18 ; epilepsy 7 ; chronic diarrhœa 7 ; typhomania 6 ; old age 5 ; congestion of brain 5 ; hemiplegia 3 ; anasarca 3 ; inflammation of brain 2 ; apoplexy 2 ; typhus fever 2 ; hypertrophy of heart 2 ; pneumonia 2 ; bronchitis, pleurisy, hydrothorax, ascites, erysipelas, scorbutus, and accidental drowning, 1 each.

So long as the circumstances controlling the population of this institution shall continue such as they are at the present time, so long must its annual records present a large bill of mortality. It is, in fact, the receptacle of the offscourings of the civilized world. Of the 371 patients received in 1855, only 78 were natives of the United States, while 293 were foreigners. Of the latter, 288 were Europeans. Ireland was represented by 178 ; the German States, including Austria and Prussia, by 68 ; England by 19 ; and eight of the other nations by smaller numbers. Some of these came with broken constitutions, many of them under the depressing influence of disappointed hopes, many with the typhoid effects of the voyage by sea still upon them, and some labouring under a combination of two or more of these vultures to vitality. There are also other causes, perhaps of minor importance, but still of sufficient magnitude to swell the sum of forces tending to a fatal issue.

The moral treatment at this institution is gradually becoming broader, more systematic, and more effective. Musical concerts, or parties, have been held from two to three times in each week. "New Year's Day, the Fourth of July, Thanksgiving, and Christmas, were appropriately observed. The oration delivered by one of the inmates, on the Fourth, is a creditable production. The reading of the Declaration of Independence, the music, and the original ode, would compare favourably with the usual ceremonies on similar occasions. About three hundred and fifty patients joined in the celebration.

"One of the most pleasant and interesting of our amusements has been the holding of 'Moot Courts.' Many could directly participate in these, either as plaintiff, defendant, counsel, judge, or juryman. The minor offences alone were tried by this supreme court of Blackwell's Island. The judge, noted for benevolence and wealth, and preferring to pay the damages rather than have any one suffer from the uncertainty of the law, gave decisions—unlike those of the city courts—satisfactory to both parties."

The reports by Dr. Fonerden, of the Maryland Hospital for the Insane, are very brief, limited almost exclusively to a short account of the changes in the patients resident, and to such subjects appertaining to the management of the hospital as are of merely local interest. The statistics of those now under review, are condensed into the subjoined table :—

| | 1853. | | | 1854. | | | 1855. | | | AGGREGATE. | | |
|---|---|---|---|---|---|---|---|---|---|---|---|---|
| | M. | W. | Tot. | M. | W. | Tot. | M. | W. | Tot. | M. | W. | Tot. |
| Patients on the 1st of January . | 68 | 62 | 130 | 57 | 60 | 117 | 56 | 63 | 119 | 68 | 62 | 130 |
| Admitted in course of the year . | 29 | 21 | 50 | 23 | 23 | 46 | 42 | 28 | 70 | 94 | 72 | 166 |
| Whole number . . . . . . | 97 | 83 | 180 | 80 | 83 | 163 | 98 | 91 | 189 | 162 | 134 | 296 |
| Discharged, including deaths . | 40 | 23 | 63 | 24 | 20 | 44 | 39 | 29 | 68 | 103 | 72 | 175 |
| Remaining, December 31 . . | 57 | 60 | 117 | 56 | 63 | 119 | 59 | 62 | 121 | — | — | — |
| Recovered . . . . . . . . | 8 | 3 | 11 | 10 | 7 | 17 | 13 | 13 | 26 | 31 | 23 | 54 |
| Died . . . . . . . . . . | 7 | 2 | 9 | 5 | 3 | 8 | 13 | 4 | 17 | 25 | 9 | 34 |

Previously to the year 1855, some cases of *mania à potu* were received at this institution ; but we are informed by the report for 1850, that such cases are not enumerated in the tabular accounts of the insane.

From the report of Dr. Stokes, Physician to the Mount Hope Institution, near Baltimore, we extract the subjoined numerical results for 1854 :—

| | Men. | Women. | Total. |
|---|---|---|---|
| Patients on the 1st of Jan., 1854 . | 45 | 87 | 132 |
| Admitted in course of the year . . | 54 | 51 | 105 |
| Whole number in course of the year . | 98 | 139 | 237 |
| Discharged, including deaths . . . | 41 | 48 | 89 |
| Remaining January 1, 1855 . . . | 56 | 91 | 147* |
| Of those discharged, there were cured | 19 | 18 | 37 |
| Died . . . . . . . . . . . | 7 | 8 | 15 |

*Causes of Death.*—Acute mania, 2 ; apoplexy, 1 ; Bright's disease, 2 ; epileptic convulsions, 2 ; puerperal mania, 2 ; exhaustive mania, 3 ; phthisis, 2 ; "gradual senile decline," 1.

"Erysipelas and dysentery prevailed to a considerable extent during the summer, but in no case did they prove fatal.

* All these figures are given as they are in the report, without an attempt to harmonize them. Of men, there were 45 at the beginning of the year, and 54 admitted ; yet the total is made 98. Of the women, the two items and the aggregate similarly disagree. It is stated, in general, that 41 males and 48 females were discharged ; yet, immediately afterwards, in giving the details of cures, improvement, deaths, &c., the number of males is made 42, and that of females 47.

"During the entire year the institution has been rather more than comfortably filled."

From the remarks upon "premature removals," we make the following extract: "Those practically familiar with the habitudes of the insane, and the motives and influences under which they act, know full well that many, who are violent, noisy, and outrageous whilst under the care of their friends, become calm and docile when subjected to the mild but firm discipline and moral treatment of an asylum. Such a change does not indicate a cessation, or even (in some instances) the mitigation of disease; it merely shows that it is held under control by the varied influences brought to bear on it. . . . Many of our inmates who are peaceful and contented, cheerfully occupied throughout the day, entering with pleasure into the amusements and recreations afforded them, or rambling at will in the grounds of the asylum, would become unhappy and unmanageable if restored to the exciting cause of their malady."

We proceed to the report for the year 1855:—

| | Men. | Women. | Total. |
|---|---|---|---|
| Patients on the 1st of January . . . | 56 | 91 | 147 |
| Admitted in the course of the year . . | 49 | 46 | 95 |
| Whole number in the course of the year | 105 | 137 | 242 |
| Discharged, including deaths . . . . | 59 | 61 | 120 |
| Remaining, Jan. 1, 1856 . . . . . . | 46 | 76 | 122 |
| Of those discharged, there were cured . | 19 | 7 | 26 |
| Died . . . . . . . . . . . . . . | 7 | 7 | 14 |

Of the patients who were discharged improved, or unimproved, forty-six belonged to the district of Columbia, and, being supported by the national government, were transferred to the Government Hospital for the Insane, near Washington.

Upon the subject of injudicious visits to patients, by their friends, Dr. Stokes says: "It is astonishing with what a reckless and criminal disregard of the most earnest representations of the injury likely to be inflicted, this course is persisted in. Thus it is that the patients' mental health and future happiness are often jeoparded by the indiscreet action of those most interested in their recovery. Strange to relate, after informing them that such a step is calculated to entail chronic insanity upon the patient for life; that its certain effect will be to protract the disorder, and thus increase and prolong the trouble and expense of his maintenance, many instances have occurred during the past year wherein they have obstinately persisted in their insane course."

The following case is related in the observations upon epileptic mania: "In a case now under treatment the person, whose attacks seldom amount to spasms, or even a distortion of the features, but in whom the loss of consciousness is complete for the time, would really seem to possess two natures. His life presents two decidedly distinct phases; the one embracing a period of a week preceding or following the attacks, during which he is suspicious, timid, apprehensive of plots to destroy him, malicious and vindictive. He is then irritable and imperious, violent and gloomy. In the other phase, in a manner normal, his character manifests itself under an entirely different aspect, exhibiting the capacities of a man in possession of good sense, and free from all extravagance."

In four cases, the abuse of opiates is alleged as the cause of the mental aberration. "Opium," remarks Dr. S., "is much more used by females than by males, and there exists abundant proof that the vicious habit of this indulgence prevails much more extensively than is supposed. From two to four ounces of laudanum a day is by no means an unfrequent allowance."

Among the facilities, and the adopted plans for moral treatment mentioned in this report, are books, music, embroidery, excursions, a saddle-pony, musical reunions, dancing, games, and newspapers. In the report for 1844, it is mentioned that the anniversaries of the Fourth of July and Christmas are appropriately observed.

The report by Dr. Stribling, of the Western Lunatic Asylum, Virginia, extends over two fiscal years :—

| | Men. | Women. | Total. |
|---|---|---|---|
| Patients in the Asylum, Oct. 1, 1853 | 217 | 160 | 377 |
| Admitted in the course of two years | 90 | 63 | 153 |
| Whole number | 307 | 223 | 530 |
| Discharged, including deaths | 81 | 61 | 142 |
| Remaining, September 30th, 1855 | 226 | 162 | 388 |
| Of those discharged, there were cured | 32 | 30 | 62 |
| Died | 36 | 22 | 58 |
| Whole number of patients admitted from July 1, 1836 | 801 | 537 | 1338 |
| Discharged, cured | 302 | 214 | 516 |
| Died | 186 | 109 | 295 |

Since the last preceding report the Asylum has been so much crowded with patients that 141 applicants were rejected. Hence Dr. Stribling requests the Board of Directors to "again invoke the attention of the General Assembly to the subject of founding another asylum for the insane," and expresses his confirmed opinion that if another institution of the kind be determined upon, it should be placed in that part of the State which is west of the Alleghany mountains.

Dr. Stribling has frequently been called from his hospital duties, by subpœna, to act as an expert in courts of law. "The

Board of Directors, perceiving the evil likely to result therefrom to the interests of the asylum, presented the subject, in their report for 1843, to the legislature, and asked, 'that this officer be released from obligation of obeying such mandates, and that he be allowed, as some other officers of the Commonwealth, to give his testimony or opinion in the usual form of deposition.' The suggestion was promptly acted upon, and a law passed to that effect." Subsequently, upon receiving a subpœna in a criminal case, he refused, under this law, to obey it. The question of the constitutionality of the law, so far as relates to criminal trials, was thereupon discussed before Judge Fulton, and he, in the language of Dr. Stribling, "sent his officer with an attachment to *coerce* my attendance. The attachment was not executed, only because, under the advice of learned and able counsel, I became satisfied that, in this case, at least, 'prudence was the better part of valour.'"

Now, in our humble opinion, Dr. Stribling and his Board of Directors were wrong in the premises. We think that no superintendent of an institution should be exempt from obedience to a subpœna, in any case, either criminal or civil, in which his opinion as an expert is important to the issue. We have few experts of the kind. They are, almost without exception, connected with the institutions for the insane. Those institutions have, or ought to have, competent assistant physicians. Thus, we believe that a law releasing the superintendents from duty before legal tribunals, would be more seriously detrimental to the cause of justice, and to the welfare of society, than useful to the inmates of the institutions over which they preside.

From the few statistics of the reports of the State Lunatic Asylum of South Carolina, we select the following:—

| | 1853. | | | 1855. | | |
|---|---|---|---|---|---|---|
| | Men. | Women. | Total. | Men. | Women. | Total. |
| Patients at the beginning of the year | ... | ... | 135 | ... | ... | 174 |
| Admitted in the course of the year | 40 | 35 | 75 | ... | ... | 62 |
| Whole number in the course of the year | ... | ... | 210 | ... | ... | 236 |
| Discharged, including deaths | ... | ... | 38 | ... | ... | 65 |
| Remaining at the end of the year | 91 | 81 | 172 | 86 | 85 | 171 |
| Discharged cured | ... | ... | 22 | ... | ... | 18 |
| Died | ... | ... | 9 | ... | ... | 31 |

In regard to the mortality in 1855, Dr. Trezevant says: "We have lost 31 patients; 15 of these, from their enfeebled state, would have died under any circumstances, but their death was hurried on by the improper accommodations of the house, and the unwholesome condition of the yard. The rest suffered from bowel complaint, then prevailing, and were the victims of our want of proper ventilation and arrangements. . . . . In dry seasons the mortality is about five per cent., but in wet it has been equal to about one in three. In the present year the bowel affections commenced with the rainy season, continued whilst it lasted, and ceased when the earth was no longer saturated with moisture."

We have carefully perused the reports before us, and find therein but little which comes within the scope of our notices, while that little is upon subjects already fully laid before our readers. The chief burthen of the reports from this institution, for several years past, consists of an exposition of the imperfections of the Asylum, and the necessity of a new one. The building is old, and imperfect in its architectural construction and arrangements. Its grounds are too limited, and are immediately surrounded by dwelling-houses of citizens of Columbia. It appears that one or more of its wards are so damp as seriously to affect the health of the inmates. The whole is so much crowded that, as stated in one of the reports, there are fifty patients more than can be properly accommodated. The heating and ventilation are bad. There are no proper arrangements of baths and water-closets. In short, judging from the reports, the whole establishment stands but as a representative of the past. It is acknowledged as such by the Regents, the Physician, and the Superintendent. This has been granted for years. The question, therefore, has been—What shall be done? We have exposition after exposition of the defects. We have suggestions for erecting additional buildings to those which now exist. We have propositions to erect an entirely new establishment upon the lands now occupied. We have argument upon argument to prove that a new structure should be erected more remote from the town. And yet the question is—What shall be done? There is a liberal appropriation yet unexpended. Different models have been presented for the new edifice. One of these is preferred and highly extolled by one party, but rejected and condemned in the strongest terms by another. And still, alas! still the question is—What shall be done? And the relic of the past, with "all its imperfections on its head," continues unmolested, and its inmates rejoice in the comforts of antiquity, because the powers that be cannot agree upon a substitute.

